# Vaccination

**Published:** 1860-11

**Authors:** 


*Vaccination after Dr. W. Husband's Method.*—Dr. D· P. Smith, in a letter from Edinburgh to the *Amer. Med. Times* of September 8, thus describes Dr. Husband's method of vaccination: "On or before the eighth day after a successful vaccination, by slightly rupturing the vesicle, and dipping therein a delicate capillary glass tube, virus sufficient to vaccinate several people ascends into the tube by virtue of the capillary attraction; then withdrawing the tube and slightly shaking it, so that the virus may be brought into the middle, both ends are in succession hermetically sealed, by being held in the flame of a candle until the end melting assumes a globular form. This process is all that is necessary to be done to procure and preserve the virus. When about to use it, all that is required is to break off both ends of the tube, and, slightly abrading the arm in one, two, or three places, to blow upon these surfaces a drop or two of the fluid. Dr. Husband assures me that nine out of every ten vaccinations 'take,' and that having employed tubes that were charged two years ago, their efficiency remains unimpaired. The tubes used are very fine, and about three inches long."—*Lancet & Observer.*

